# Discovery of urine biomarkers for lupus nephritis via quantitative and comparative proteome analysis

**DOI:** 10.1002/ctm2.638

**Published:** 2021-11-11

**Authors:** Oh Chan Kwon, Eun‐Ju Lee, Jeonghun Yeom, Seokchan Hong, Chang‐Keun Lee, Bin Yoo, Min‐Chan Park, Kyunggon Kim, Yong‐Gil Kim

**Affiliations:** ^1^ Division of Rheumatology Department of Internal Medicine Yonsei University College of Medicine Seoul Korea; ^2^ Division of Rheumatology Department of Internal Medicine University of Ulsan College of Medicine, Asan Medical Center Seoul Korea; ^3^ Convergence Medicine Research Center Asan Institution for Life Science Asan Medical Center Seoul Korea; ^4^ Department of Biomedical Sciences University of Ulsan College of Medicine Seoul Korea; ^5^ Bio‐Medical Institute of Technology Asan Medical Center Seoul Korea

To the Editor:

Lupus nephritis (LN) is the leading cause of morbidity and mortality in patients with systemic lupus erythematosus (SLE).^1^ Noninvasively obtainable biomarkers for LN could greatly improve early LN detection. In this study, we found that urine alpha‐1‐acid glycoprotein (ORM1) is a promising biomarker for early LN detection. Furthermore, with regard to histologic features, urine haemoglobin subunit delta (HBD) accurately discriminated proliferative LN from nonproliferative LN and correlated with activity index.

Using sequential window acquisition of all theoretical mass spectra combined with liquid chromatography (SWATH LC–MS) platform, a novel mass spectrometry‐based proteome analysis,^2^ we screened potential biomarker candidates in urine samples from 20 healthy controls (HCs) and 39 patients with SLE with or without newly diagnosed LN (n‐LN). All patients with SLE met the 2012 Systemic Lupus International Collaborating Clinics classification criteria for SLE.^3^ Sample preparation and in‐house urine proteome spectral library generation were performed as described previously.^2^ In our spectral library, 2323 proteins and 8371 peptides were identified using data‐dependent acquisition analysis. A total of 1157 protein groups (581 ± 31, 506 ± 25 and 457 ± 22 in HCs, patients with SLE without nephritis, and patients with n‐LN, respectively) were quantified from individual samples using an in‐house spectral library. Among the 1157 protein groups, 143 protein groups (false discovery rate ≤.05, log2 fold change ±1) were increased in patients with SLE without nephritis than those in HCs (Figure 1A), and 67 protein groups were increased in patients with n‐LN than those in patients with SLE without nephritis (Figure 1B). Among these protein groups, 23 protein groups were identified to be common between the two comparative analyses. Among these 23 urine proteins, five proteins (ORM1, antithrombin‐III [SERPINC1], ceruloplasmin [CP], haemoglobin subunit beta [HBB] and HBD) were selected for enzyme‐linked immunosorbent assay (ELISA)‐based validation. The characteristics between patients with SLE without nephritis (*n* = 22) and patients with n‐LN (*n* = 17) included in the validation study were compared (Table 1). Importantly, three patients with n‐LN (17.3%) had nonsignificant proteinuria (urine protein/creatinine ratio <500 mg/g). Therefore, the urine biomarkers identified herein could be useful in early LN detection, even before significant proteinuria develops. The ELISA results revealed that urine ORM1, SERPINC1, CP and HBD, normalised to urine creatinine, were significantly upregulated in patients with n‐LN than in patients with SLE without nephritis (Figure 1C–G). Similar results were observed with urine ORM1, SERPINC1, CP and HBD, not normalised to urine creatinine (Figure S1).

**FIGURE 1:**
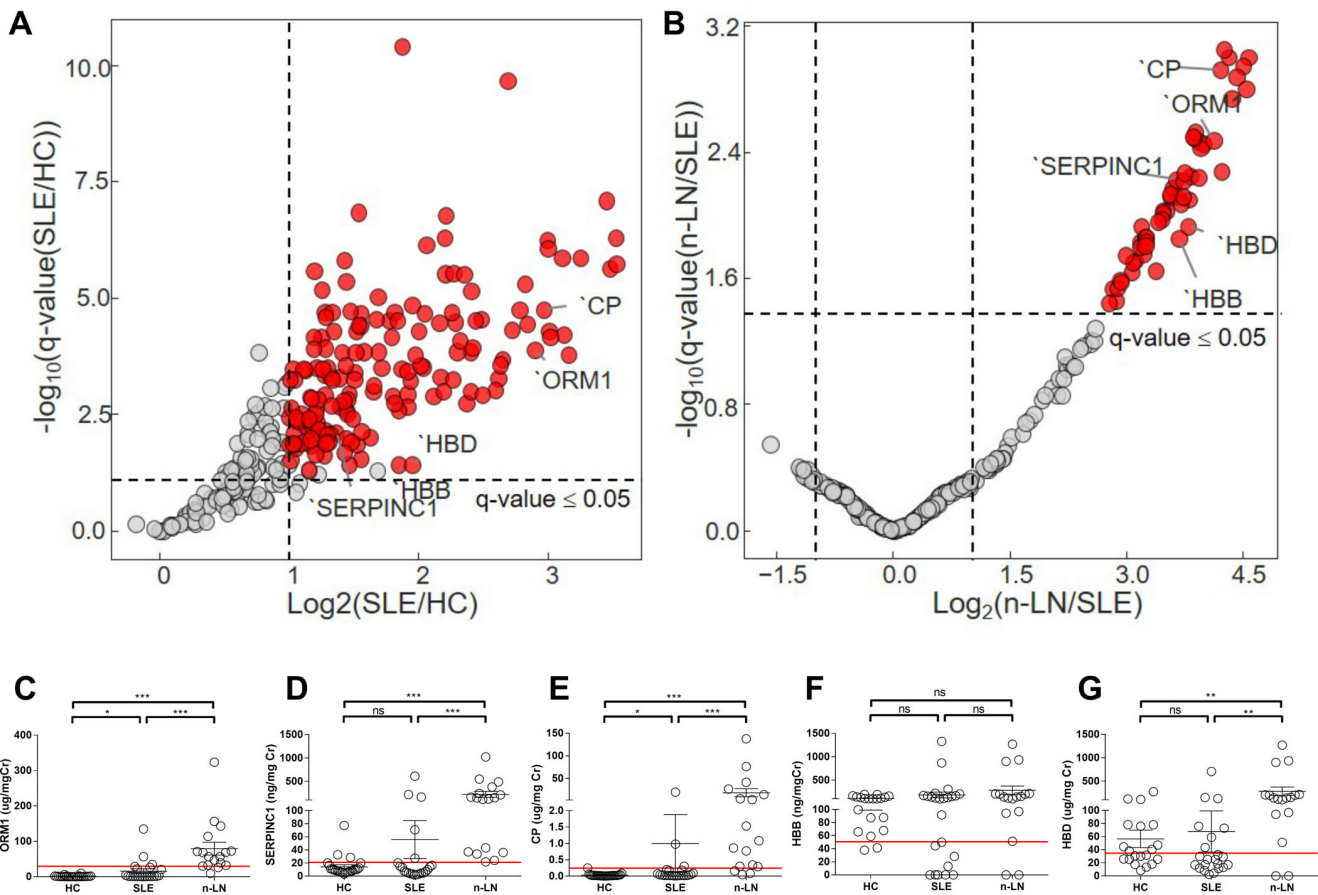
Comparative analysis of proteome in HCs, patients with SLE without nephritis, and patients with n‐LN, and ELISA‐based validation. Volcano plot of protein expression (proteins ranked according to their *q*‐value; log10 scale, *y*‐axis) and their relative abundance ratio (log2 fold change, *x*‐axis) between (A) HCs versus SLE without nephritis and (B) SLE without nephritis versus n‐LN. ELISA‐based validation of (C) urine ORM1, (D) urine SERPINC1, (E) urine CP, (F) urine HBB, and (G) urine HBD, normalised to urine creatinine. Red dots in (A) and (B) indicate differentially expressed proteins (FDR: ≤.05, log2 fold change ±1) between groups. Horizontal dotted lines indicate *q*‐value of .05 and vertical dotted lines indicate log fold change of 2 in (A) and (B). *t*‐Test using the Benjamini–Hochberg FDR (.05 cutoff) was performed to find statistically significant differences between samples in (A) and (B). Values presented in (C)–(G) are the mean ± SEM. Red lines in (C)–(G) indicate the optimal cutoff value (i.e., value where the Youden's index is maximum) of each urine protein for discriminating n‐LN from SLE without nephritis. The cutoff value of each urine protein was as follows: urine ORM1, 29.61 μg/mgCr (sensitivity: .882, specificity: .864, likelihood ratio: 6.47); urine SERPINC1, 20.96 ng/mgCr (sensitivity: 1.000, specificity: .773, likelihood ratio: 4.40); urine CP, 0.24 μg/mgCr (sensitivity: .824, specificity: .864, likelihood ratio: 6.04); urine HBB, 50.66 ng/mgCr (sensitivity: .882, specificity: .375, likelihood ratio: 1.41); and urine HBD, 34.71 μg/mgCr (sensitivity: .765, specificity: .682, likelihood ratio: 2.40). Kruskal–Wallis test was used to compare the three groups in (C)–(G), and Mann–Whitney *U* tests for multiple comparisons. *p < .05, **p < .01, ***p < .001. CP, ceruloplasmin; ELISA, enzyme‐linked immunosorbent assay; HBB, haemoglobin subunit beta; HBD, haemoglobin subunit delta; HCs, healthy controls; n‐LN, newly diagnosed lupus nephritis; ns, not significant; ORM1, alpha‐1‐acid glycoprotein; SERPINC1, antithrombin‐III; SLE, systemic lupus erythematosus

**TABLE 1:** Comparison of baseline characteristics between patients with SLE with and without nephritis

	**SLE without nephritis(*N* = 22)**	**n‐LN(*N* = 17)**	***p*‐Value**
Age, years, median (IQR)	51.0 (37.5–56.3)	43.0 (23.0–49.5)	.036
Female sex, *n* (%)	18 (81.8)	14 (82.4)	>.999
Disease duration, months, median (IQR)	32.5 (17.1–57.8)	36.9 (1.9–78.1)	.790
SLE manifestations, *n* (%)			
Mucocutaneous	5 (22.7)	3 (17.6)	>.999
Musculoskeletal	8 (36.4)	3 (17.6)	.288
Serositis	2 (9.1)	0 (0.0)	.495
Neuropsychiatric	2 (9.1)	2 (11.8)	>.999
Hematologic	3 (13.6)	3 (17.6)	>.999
C3, mg/dl, median (IQR)	97.6 (76.7–110.8)	53.9 (43.0–91.9)	.005
C4, mg/dl, median (IQR)	20.8 (12.9–25.6)	9.4 (5.5–23.0)	.048
Anti‐dsDNA Ab, IU/ml, median (IQR)	7.5 (5.4–19.8)	121.0 (5.7–377.0)	.021
Positive anti‐Sm Ab, *n* (%)	4 (18.2)	5 (31.3)	.450
Positive anti‐Ro Ab, *n* (%)	10 (45.5)	9 (56.3)	.511
Positive anti‐La Ab, *n* (%)	2 (9.1)	3 (18.8)	.632
Positive anti‐U1RNP Ab, *n* (%)	5 (22.7)	5 (31.3)	.713
Anaemia, *n* (%)	8 (36.4)	10 (58.8)	.163
ESR, mm/h, median (IQR)	19.0 (12.0–33.8)	29.0 (18.0–36.0)	.267
CRP, mg/dl, median (IQR)	0.15 (0.10–0.23)	0.18 (0.10–0.65)	.878
Serum creatinine, mg/dl, median (IQR)	0.60 (0.55–0.76)	0.72 (0.56–0.99)	.098
UPCR, mg/g, median (IQR)	92.2 (64.8–175.0)	970.9 (585.6–2328.1)	<.001
UPCR <500 mg/g, *n* (%)	22 (100.0)	3 (17.6)	<.001
Urine RBC ≥5/HPF, *n* (%)	3 (13.6)	4 (23.5)	.677
Urine WBC ≥5/HPF, *n* (%)	2 (9.1)	5 (29.4)	.205
SLEDAI, median (IQR)	5.0 (2.0–8.0)	12.0 (7.0–12.0)	.003
Renal SLEDAI, median (IQR)	0.0 (0.0–0.0)	4.0 (4.0–8.0)	<.001
Extra‐renal SLEDAI, median (IQR)	4.0 (2.0–8.0)	4.0 (1.0–7.0)	.989
Medications, *n* (%)			
Hydroxychloroquine	21 (95.5)	14 (82.4)	.300
Mycophenolate mofetil	1 (4.5)	2 (11.8)	.570
Azathioprine	1 (4.5)	1 (5.9)	>.999
Methotrexate	6 (27.3)	0 (0.0)	.027
Tacrolimus	1 (4.5)	0 (0.0)	>.999
Glucocorticoid	11 (50.0)	12 (70.6)	.195
Renal histology			
ISN/RPS class, *n* (%)			
I	N/A	1 (5.9)	N/A
II		5 (29.4)	
III		4 (23.5)	
IV		1 (5.9)	
V		5 (29.4)	
III + V		1 (5.9)	
Activity index, median (IQR)	N/A	1.0 (1.0–4.0)	N/A
Chronicity index, median (IQR)	N/A	2.0 (1.0–3.5)	N/A

Abbreviations: anti‐dsDNA Ab, anti‐double stranded DNA antibody; CRP, C‐reactive protein; ESR, erythrocyte sedimentation rate; HPF, high‐power field; ISN/RPS, International Society of Nephrology/Renal Pathology Society; n‐LN, newly diagnosed lupus nephritis; RBC, red blood cell; SLE, systemic lupus erythematosus; SLEDAI, systemic lupus erythematosus disease activity index; UPCR, urine protein creatinine ratio; WBC, white blood cell.

The accuracy of the urine biomarkers and traditional serologic factors (C3, C4 and anti‐dsDNA antibody)^4^ for discriminating n‐LN from SLE without nephritis was evaluated using receiver operating characteristic (ROC) analysis (Figure 2A–I). The area under the curves (AUCs) of urine ORM1, SERPINC1, CP and HBD were .914, .874, .896 and .757, respectively (Figure 2A–D). Urine ORM1, SERPINC1 and CP had higher AUCs than the traditional serologic factors (Figure 2E–G: C3, .759; C4, .689; and anti‐dsDNA antibody, .715). Further, we evaluated whether combinations of variables enhanced discriminatory ability. Two composite parameters were assessed: combination of all four urine biomarkers (ORM1 + SERPINC1 + CP + HBD) and combination of factors selected using logistic regression analysis with stepwise forward selection (C3 + urine ORM1). ORM1 + SERPINC1 + CP + HBD had an AUC of .901 and C3 + urine ORM1 had an AUC of .920 (Figure 2H,I). Although the AUC was the highest with C3 + urine ORM1, it was only slightly higher than that of urine ORM1 alone. This suggests that urine ORM1 accurately distinguishes patients with n‐LN from patients with SLE without nephritis. ORM, a member of the acute phase protein family, activates monocytes, induces T‐cell proliferation, and promotes the secretion of proinflammatory cytokines.^5^ However, its biological role in LN is poorly understood. Although its pathogenic role in LN remains unclear, previous study has highlighted urine ORM1 as a biomarker for active LN.^6^ Our present finding adds to the previous knowledge that urine ORM1 is not only a biomarker for active LN, but also a promising biomarker for early LN detection.

**FIGURE 2:**
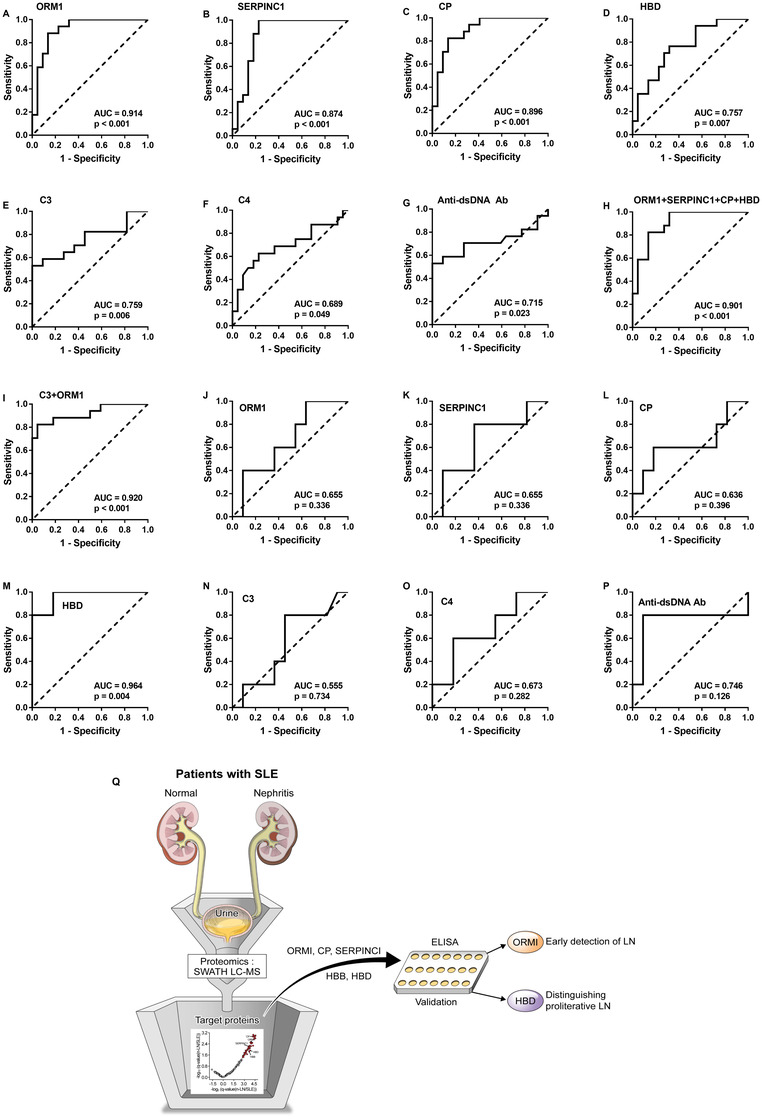
Receiver operating characteristic curves for discriminating n‐LN from SLE without nephritis using (A) urine ORM1, (B) urine SERPINC1, (C) urine CP, (D) urine HBD, (E) serum C3, (F) serum C4, (G) serum anti‐dsDNA Ab, (H) combination of urine ORM1, SERPINC1, CP and HBD, and (I) combination of serum C3 and urine ORM1. Receiver operating characteristic curves for discriminating proliferative LN from nonproliferative LN using (J) urine ORM1, (K) urine SERPINC1, (L) urine CP, (M) urine HBD, (N) serum C3, (O) serum C4, and (P) serum anti‐dsDNA Ab. (Q) Summary of the present SWATH LC–MS‐based biomarker study. AUC, area under the curve; CP, ceruloplasmin; ELISA, enzyme‐linked immunosorbent assay; HBD, haemoglobin subunit delta; HBB, haemoglobin subunit beta; LN, lupus nephritis; ORM1, alpha‐1‐acid glycoprotein; SERPINC1, antithrombin‐III; SLE, systemic lupus erythematosus; SWATH LC–MS, sequential window acquisition of all theoretical mass spectra combined with liquid chromatography

Next, additional ROC analyses were performed to assess the ability of the urine biomarkers for distinguishing proliferative LN (class III or IV according to International Society of Nephrology/Renal Pathology Society [ISN/RPS] class) from nonproliferative LN (class I, II or V according to ISN/RPS class) (Figure 2J–P). Urine HBD had a high accuracy (AUC = .964) in differentiating proliferative LN from nonproliferative LN (Figure 2M). As differentiating proliferative LN from nonproliferative LN is important in the perspective of treatment strategy,^7^ this discriminatory ability of urine HBD has clinical implication. We also assessed the correlation between urine biomarkers and activity/chronicity indices (Table 2). Of the four urine biomarkers, only urine HBD was significantly positively correlated with the activity index (rho = .549, *p* = .024). The correlation was stronger when urine HBD was used in combination with other urine proteins (ORM1 + SERPINC1 + CP + HBD: rho = .727, *p* = .001). However, none of the urine biomarkers showed correlation with chronicity index. The activity index after treatment is an important prognostic factor of LN.^8^ Clinical renal parameters do not accurately reflect the activity index,^9^ and it is difficult to presume the activity index without performing renal biopsies. Nevertheless, renal biopsy is an invasive procedure and is often difficult to perform repeatedly. In such cases, urine HBD could be useful for speculating the activity index. HBD has been suggested to be a potential biomarker for early diabetic kidney disease in a recent proteomics study.^10^ The possible mechanism underlying urine HBD in diabetic kidney disease pathogenesis is thought to be inflammation and oxidative stress.^10^ However, its association with LN has not been known to date. We provide the first evidence that it could be used as a biomarker to reflect histologic information in patients with LN.

**TABLE 2:** Correlation between potential urine biomarkers for LN and pathologic activity/chronicity index

	**Activity index**			**Chronicity index**		
	**Rho (95% CI)**	***R* ^2^**	***p*‐Value**	**Rho (95% CI)**	*R* ^2^	*p*‐Value
Urine CP	−.065 (−.540, .442)	.004	.804	−.258 (−.666, .269)	.067	.315
Urine SERPINC1	.307 (−.218, .695)	.094	.229	.218 (−.308, .642)	.048	.398
Urine ORM1	.172 (−.350, .613)	.030	.506	.119 (−.397, .578)	.014	.647
Urine HBD	.549 (.077, .820)	.301	.024	−.024 (−.510, .474)	.001	.929
Combination^a^	.727 (.366, .898)	.529	.001	.079 (−.430, .550)	.006	.763

*Note*: Correlations analysed using Spearman's correlation test.

Abbreviations: CP, ceruloplasmin; HBD, haemoglobin subunit delta; ORM1, alpha‐1‐acid glycoprotein; SERPINC1, antithrombin‐III.

^a^ Urine ORM1 + SERPINC1 + CP + HBD.

In summary (Figure 2Q), although limited by the small sample size and lack of replication in an independent cohort, we showed that urine ORM1 can accurately detect LN, even in the early disease where significant amount of proteinuria has yet developed. In addition, urine HBD had an excellent accuracy in differentiating proliferative LN from nonproliferative LN and correlated with activity index. Therefore, these urine biomarkers may provide important information, particularly when renal biopsies are unavailable. Although these findings are promising, further studies with more patients are warranted for a powerful conclusion.

## ETHICS APPROVAL AND CONSENT TO PARTICIPATE

The Institutional Review Board of Asan Medical Center in Seoul, South Korea, approved the study (IRB No. 2013‐0405). Written informed consent was obtained from all patients.

## CONFLICT OF INTEREST

The authors declare that there is no conflict of interest.

## Supporting information

SUPPORTING INFORMATION
**Supporting Figure S1** ELISA of (A) urine ORM1, (B) urine SERPINC1, (C) urine CP, (D) urine HBB, and (E) urine HBD, not normalised to urine creatinine. Values presented are the mean ± SEM. Kruskal–Wallis test was used to compare the three groups, and Mann–Whitney *U* tests for multiple comparisons. *p < .05, **p < .01, ***p < .001. HCs, healthy controls; SLE, systemic lupus erythematosus; n‐LN, newly diagnosed lupus nephritis; ELISA, enzyme‐linked immunosorbent assay; ORM1, alpha‐1‐acid glycoprotein; SERPINC1, antithrombin‐III; CP, ceruloplasmin; HBB, haemoglobin subunit beta; HBD, haemoglobin subunit delta; ns, not significant

Supporting information
