# Supplementary material for: Discovery of urine biomarkers for lupus nephritis via quantitative and comparative proteome analysis
Source: Clin Transl Med. 2021 Nov 11;11(11):e638. doi: 10.1002/ctm2.638 (PMC8582290; doi:10.1002/ctm2.638)
Supplement: Supplementary file 1 — SUPPORTING INFORMATION Supporting Figure S1 ELISA of (A) urine ORM1, (B) urine SERPINC1, (C) urine CP, (D) urine HBB, and (E) urine HBD, not normalised to urine creatinine. Values presented are the mean ± SEM. Kruskal–Wallis test was used to compare the three groups, and Mann–Whitney U tests for multiple comparisons. *p < .05, **p < .01, ***p < .001. HCs, healthy controls; SLE, systemic lupus erythematosus; n‐LN, newly diagnosed lupus nephritis; ELISA, enzyme‐linked immunosorbent assay; ORM1, alpha‐1‐acid glycoprotein; SERPINC1, antithrombin‐III; CP, ceruloplasmin; HBB, haemoglobin subunit beta; HBD, haemoglobin subunit delta; ns, not significant [file CTM2-11-e638-s001.docx]

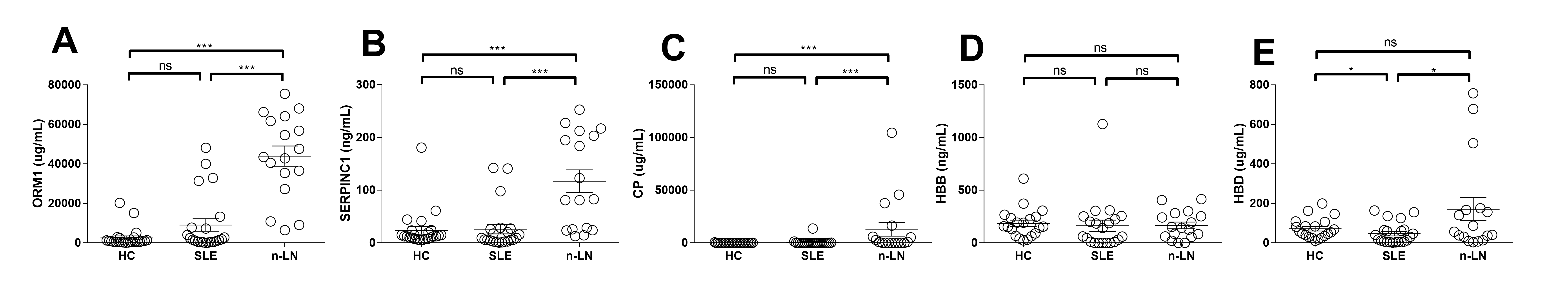


**Supplementary Figure 1.** ELISA of **(A)** urine ORM1, **(B)** urine SERPINC1, **(C)** urine CP, **(D**) urine HBB, and **(E)** urine HBD, not normalized to urine creatinine. Values presented are the mean±SEM. Kruskal-Wallis test was used to compare the three groups, and Mann-Whitney U tests for multiple comparisons. * p<0.05, ** p<0.01, *** p<0.001.

HCs, healthy controls; SLE, systemic lupus erythematosus; n-LN, newly diagnosed lupus nephritis; ELISA, enzyme-linked immunosorbent assay; ORM1, alpha-1-acid glycoprotein; SERPINC1, antithrombin-III; CP, ceruloplasmin; HBB, haemoglobin subunit beta; HBD, haemoglobin subunit delta; ns, not significant.
