# Supplementary material for: Discovery of urine biomarkers for lupus nephritis via quantitative and comparative proteome analysis
Source: Clin Transl Med. 2021 Nov 11;11(11):e638. doi: 10.1002/ctm2.638 (PMC8582290; doi:10.1002/ctm2.638)
Supplement: Supplementary file 2 — Supporting information [file CTM2-11-e638-s002.docx]

**Supplementary Table 1.** List of the 23 proteins identified in the SWATH LC-MS analysis

| Protein group | Gene | Description | Function | Biomarkers for other diseases | Commercial ELISA kit | | Supplementary References |
| --- | --- | --- | --- | --- | --- | --- | --- |
| P43652 | AFM | Afamin | A plasma vitamin E-binding glycoprotein. Facilitates vitamin E transport across the blood-brain barrier. | Ovarian cancer, cervical cancer | Available | | S1-S3 |
| P02763 | ORM1 | Alpha-1-acid glycoprotein 1 | One of the major acute phase proteins, which serum concentration increases in response to systemic tissue injury, inflammation or infection. | Diabetic renal disease | Available | | S4,S5 |
| P19652 | ORM2 | Alpha-1-acid glycoprotein 2 | One of the major acute phase proteins, which serum concentration increases in response to systemic tissue injury, inflammation or infection. | Diabetic renal disease | Available | | S4,S5 |
| P01011 | SERPINA3 | Alpha-1-antichymotrypsin | A major plasma serine proteinase inhibitor. | Alzheimer’s disease | Available | | S6,S7 |
| P01009 | SERPINA1 | Alpha-1-antitrypsin | A prominent protease inhibitor. | Hepatitis B | Available | | S8 |
| P04217;P04217-2 | A1BG | Alpha-1B-glycoprotein | Function yet to be elucidated. | Cervical cancer, pediatric steroid-resistant nephrotic syndrome | Available | | S3,S9 |
| P04217 | A1BG | Alpha-1B-glycoprotein | Function yet to be elucidated. | Cervical cancer, pediatric steroid-resistant nephrotic syndrome | Available | | S3,S9 |
| P01008 | SERPINC1 | Antithrombin-III | The primary inhibitor of plasma protease coagulation proteins. | Neonatal sepsis | Available | | S10,S11 |
| P00915 | CA1 | Carbonic anhydrase 1 | A zinc metalloenzyme that catalyzes the reversible hydration of carbon dioxide to bicarbonate. | Non-small cell lung cancer, prostate cancer | Available | | S12,S13 |
| P00450 | CP | Ceruloplasmin | A critical physiologic role in controlling the rate of iron efflux from cells with mobilizable iron. | Cervical cancer | Available | | S3,S14 |
| P08185 | SERPINA6 | Corticosteroid-binding globulin | A plasma glycoprotein that binds ~90% of circulating glucocorticoids, and regulates their bioavailability in target tissues. | Onset and severity of inflammation in rats | Available | | S15,S16 |
| P43246 | MSH2 | DNA mismatch repair protein Msh2 | A protein that plays an essential role in repairing DNA. | Lynch syndrome, ovarian cancer | Available | | S17,S18 |
| Q8NC42 | RNF149 | E3 ubiquitin-protein ligase RNF149 | Involved in control of gene transcription, translation, cell adhesion, cytoskeletal organization or epithelial development. | Non-small cell lung cancer | Available | | S19 |
| P00738 | HP | Haptoglobin | A plasma glycoprotein that binds free hemoglobin and plays a critical role in tissue protection and the prevention of oxidative damage. | Various forms of malignant neoplasms | Available | | S20 |
| Q96RW7 | HMCN1 | Hemicentin-1 | An extracelluar matrix protein that functions in cell-to-cell anchoring and is vital for maintaining cell adherence to tissue and organ basement membrane. | Age-related macular degeneration, podocyte dysfunction | Available | | S21-S23 |
| P69905 | HBA1 | Hemoglobin subunit alpha | A protein that constitutes hemoglobin A. | Ovarian cancer | Available | | S24 |
| P68871 | HBB | Hemoglobin subunit beta | A protein that constitutes hemoglobin A. | Ovarian cancer | Available | | S24 |
| P02042 | HBD | Hemoglobin subunit delta | A protein that constitutes hemoglobin A2. | Early diabetic kidney disease | Available | | S25 |
| P02750 | LRG1 | Leucine-rich alpha-2-glycoprotein | Suggested to be associated with inflammatory responses and neutrophilic differentiation as well as cellular responses to the profibrotic cytokine transforming growth factor-β. | Active tuberculosis, Disease activity of rheumatoid arthritis and inflammatory bowel diseases, heart failure, pancreatic cancer | Available | | S26-S30 |
| Q15293 | RCN1 | Reticulocalbin-1 | A calcium-binding protein stored in endoplasmic reticulum. | Renal cell carcinoma | Available | | S31,S32 |
| P02787 | TF | Serotransferrin | Bind and transport iron throughout the body. | Cholangiocarcinoma | Available | | S33 |
| P02768 | ALB | Serum albumin | An abundant multifunctional non-glycosylated, negatively charged plasma protein, with ascribed ligand-binding and transport properties, antioxidant functions, and enzymatic activities. Is responsible for maintaining colloid osmotic pressure and may influence microvascular integrity and aspects of the inflammatory pathway, including neutrophil adhesion and the activity of cell signaling moieties. | Nutritional status, inflammation, hepatic synthesis capacity, increased mortality from cancers, respiratory diseases, and heart failure | Available | | S34-S39 |
| P25311 | AZGP1 | Zinc-alpha-2-glycoprotein | Stimulates lipolysis through stimulation of adenylate cyclase in a guanosine triphosphate-dependent process via binding through the β3 adrenoreceptor. | Diabetic nephropathy | Available | S40-S42 | |

**Supplementary References**

S1. Dieplinger H, Dieplinger B. Afamin--A pleiotropic glycoprotein involved in various disease states. *Clin Chim Acta.* 2015;446:105-110.

S2. Jackson D, Craven RA, Hutson RC, et al. Proteomic profiling identifies afamin as a potential biomarker for ovarian cancer. *Clin Cancer Res.* 2007;13(24):7370-7379.

S3. Jeong DH, Kim HK, Prince AE, et al. Plasma proteomic analysis of patients with squamous cell carcinoma of the uterine cervix. *J Gynecol Oncol.* 2008;19(3):173-180.

S4. Gomes MB, Nogueira VG. Acute-phase proteins and microalbuminuria among patients with type 2 diabetes. *Diabetes Res Clin Pract.* 2004;66(1):31-39.

S5. Fournier T, Medjoubi NN, Porquet D. Alpha-1-acid glycoprotein. *Biochim Biophys Acta.* 2000;1482(1-2):157-171.

S6. Forsyth S, Horvath A, Coughlin P. A review and comparison of the murine alpha1-antitrypsin and alpha1-antichymotrypsin multigene clusters with the human clade A serpins. *Genomics.* 2003;81(3):336-345.

S7. Padmanabhan J, Levy M, Dickson DW, Potter H. Alpha1-antichymotrypsin, an inflammatory protein overexpressed in Alzheimer's disease brain, induces tau phosphorylation in neurons. *Brain.* 2006;129(Pt 11):3020-3034.

S8. Tan XF, Wu SS, Li SP, Chen Z, Chen F. Alpha-1 antitrypsin is a potential biomarker for hepatitis B. *Virol J.* 2011;8:274.

S9. Piyaphanee N, Ma Q, Kremen O, et al. Discovery and initial validation of α 1-B glycoprotein fragmentation as a differential urinary biomarker in pediatric steroid-resistant nephrotic syndrome. *Proteomics Clin Appl.* 2011;5(5-6):334-342.

S10. Pengo V, Guerra C, Cartei G, Fiorentino M. Behavior of antithrombin III, determined as protein concentration and biologic activity, in subjects with neoplastic disease. *Tumori.* 1982;68(3):205-209.

S11. Samra N, AlGhwass M, Elgawhary S, et al. Serum Level of Antithrombin III (ATIII) Could Serve as a Prognostic Biomarker in Neonatal Sepsis. *Fetal Pediatr Pathol.* 2019;38(4):290-298.

S12. Wang DB, Lu XK, Zhang X, Li ZG, Li CX. Carbonic anhydrase 1 is a promising biomarker for early detection of non-small cell lung cancer. *Tumour Biol.* 2016;37(1):553-559.

S13. Takakura M, Yokomizo A, Tanaka Y, et al. Carbonic anhydrase I as a new plasma biomarker for prostate cancer. *ISRN Oncol.* 2012;2012:768190.

S14. Hellman NE, Gitlin JD. Ceruloplasmin metabolism and function. *Annu Rev Nutr.* 2002;22:439-458.

S15. Lin HY, Muller YA, Hammond GL. Molecular and structural basis of steroid hormone binding and release from corticosteroid-binding globulin. *Mol Cell Endocrinol.* 2010;316(1):3-12.

S16. Hill LA, Bodnar TS, Weinberg J, Hammond GL. Corticosteroid-binding globulin is a biomarker of inflammation onset and severity in female rats. *J Endocrinol.* 2016;230(2):215-225.

S17. Pande M, Wei C, Chen J, et al. Cancer spectrum in DNA mismatch repair gene mutation carriers: results from a hospital based Lynch syndrome registry. *Fam Cancer.* 2012;11(3):441-447.

S18. Pennington KP, Swisher EM. Hereditary ovarian cancer: beyond the usual suspects. *Gynecol Oncol.* 2012;124(2):347-353.

S19. Fan Q, Wang Q, Cai R, Yuan H, Xu M. The ubiquitin system: orchestrating cellular signals in non-small-cell lung cancer. *Cell Mol Biol Lett.* 2020;25:1.

S20. Naryzhny SN, Legina OK. [Haptoglobin as a biomarker]. *Biomed Khim.* 2021;67(2):105-118.

S21. Xu X, Xu M, Zhou X, et al. Specific structure and unique function define the hemicentin. *Cell Biosci.* 2013;3(1):27.

S22. Toffoli B, Zennaro C, Winkler C, et al. Hemicentin 1 influences podocyte dynamic changes in glomerular diseases. *Am J Physiol Renal Physiol.* 2018;314(6):F1154-f1165.

S23. Schultz DW, Klein ML, Humpert AJ, et al. Analysis of the ARMD1 locus: evidence that a mutation in HEMICENTIN-1 is associated with age-related macular degeneration in a large family. *Hum Mol Genet.* 2003;12(24):3315-3323.

S24. Woong-Shick A, Sung-Pil P, Su-Mi B, et al. Identification of hemoglobin-alpha and -beta subunits as potential serum biomarkers for the diagnosis and prognosis of ovarian cancer. *Cancer Sci.* 2005;96(3):197-201.

S25. Golea-Secara A, Munteanu C, Sarbu M, et al. Urinary proteins detected using modern proteomics intervene in early type 2 diabetic kidney disease - a pilot study. *Biomark Med.* 2020;14(16):1521-1536.

S26. Fujimoto M, Matsumoto T, Serada S, et al. Leucine-rich alpha 2 glycoprotein is a new marker for active disease of tuberculosis. *Sci Rep.* 2020;10(1):3384.

S27. Serada S, Fujimoto M, Ogata A, et al. iTRAQ-based proteomic identification of leucine-rich alpha-2 glycoprotein as a novel inflammatory biomarker in autoimmune diseases. *Ann Rheum Dis.* 2010;69(4):770-774.

S28. Shinzaki S, Matsuoka K, Iijima H, et al. Leucine-rich Alpha-2 Glycoprotein is a Serum Biomarker of Mucosal Healing in Ulcerative Colitis. *J Crohns Colitis.* 2017;11(1):84-91.

S29. Watson CJ, Ledwidge MT, Phelan D, et al. Proteomic analysis of coronary sinus serum reveals leucine-rich α2-glycoprotein as a novel biomarker of ventricular dysfunction and heart failure. *Circ Heart Fail.* 2011;4(2):188-197.

S30. Furukawa K, Kawamoto K, Eguchi H, et al. Clinicopathological Significance of Leucine-Rich α2-Glycoprotein-1 in Sera of Patients With Pancreatic Cancer. *Pancreas.* 2015;44(1):93-98.

S31. Giribaldi G, Barbero G, Mandili G, et al. Proteomic identification of Reticulocalbin 1 as potential tumor marker in renal cell carcinoma. *J Proteomics.* 2013;91:385-392.

S32. Ozawa M, Muramatsu T. Reticulocalbin, a novel endoplasmic reticulum resident Ca(2+)-binding protein with multiple EF-hand motifs and a carboxyl-terminal HDEL sequence. *J Biol Chem.* 1993;268(1):699-705.

S33. Jamnongkan W, Lebrilla CB, Barboza M, et al. Discovery of Serotransferrin Glycoforms: Novel Markers for Diagnosis of Liver Periductal Fibrosis and Prediction of Cholangiocarcinoma. *Biomolecules.* 2019;9(10).

S34. Quinlan GJ, Martin GS, Evans TW. Albumin: biochemical properties and therapeutic potential. *Hepatology.* 2005;41(6):1211-1219.

S35. Sullivan DH, Sun S, Walls RC. Protein-energy undernutrition among elderly hospitalized patients: a prospective study. *Jama.* 1999;281(21):2013-2019.

S36. Kaysen GA, Dubin JA, Müller HG, Mitch WE, Rosales LM, Levin NW. Relationships among inflammation nutrition and physiologic mechanisms establishing albumin levels in hemodialysis patients. *Kidney Int.* 2002;61(6):2240-2249.

S37. Fuhrman MP, Charney P, Mueller CM. Hepatic proteins and nutrition assessment. *J Am Diet Assoc.* 2004;104(8):1258-1264.

S38. Corti MC, Guralnik JM, Salive ME, Sorkin JD. Serum albumin level and physical disability as predictors of mortality in older persons. *Jama.* 1994;272(13):1036-1042.

S39. Ancion A, Allepaerts S, Robinet S, Oury C, Pierard LA, Lancellotti P. Serum albumin level and long-term outcome in acute heart failure. *Acta Cardiol.* 2019;74(6):465-471.

S40. Elsheikh M, Elhefnawy KA, Emad G, Ismail M, Borai M. Zinc alpha 2 glycoprotein as an early biomarker of diabetic nephropathy in patients with type 2 diabetes mellitus. *J Bras Nefrol.* 2019;41(4):509-517.

S41. Wang Y, Li YM, Zhang S, Zhao JY, Liu CY. Adipokine zinc-alpha-2-glycoprotein as a novel urinary biomarker presents earlier than microalbuminuria in diabetic nephropathy. *J Int Med Res.* 2016;44(2):278-286.

S42. Hassan MI, Waheed A, Yadav S, Singh TP, Ahmad F. Zinc alpha 2-glycoprotein: a multidisciplinary protein. *Mol Cancer Res.* 2008;6(6):892-906.
